# An Uninformative Truth: The Logic of Amarin’s Off-Label Promotion

**DOI:** 10.1371/journal.pmed.1001978

**Published:** 2016-03-15

**Authors:** Spencer Phillips Hey, Aaron S. Kesselheim

**Affiliations:** 1 The Program On Regulation, Therapeutics, And Law (PORTAL), Division of Pharmacoepidemiology and Pharmacoeconomics, Department of Medicine, Brigham and Women’s Hospital and Harvard Medical School, Boston, Massachusetts, United States of America; 2 The Harvard Center for Bioethics, Harvard Medical School, Boston, Massachusetts, United States of America

## Abstract

Spencer Phillips Hey and Aaron Kesselheim propose that informativeness—asserting scientific facts—rather than truthfulness ought to be the standard for regulating commercial speech about pharmaceuticals.

**Summary Points**

- The United States Food and Drug Administration (FDA) generally does not permit pharmaceutical manufacturers to promote their products for non-FDA-approved (“off-label”) indications. A lawsuit filed by Amarin Pharmaceutical in May 2015 sought permission to distribute off-label statements relating to its product Vascepa, because it argued that those statements were “truthful and non-misleading” and that the First Amendment’s protection of commercial speech gave it the right to make those statements. In August 2015, a federal court agreed with Amarin.
- We argue that Amarin’s argument is faulty, because the so-called “truthful and non-misleading” statement is a tautology—that is, it is only truthful because of its logical construction—not because it is consistent with the state of scientific evidence. Since the off-label marketing statement is actually misleading when viewed from this perspective, the federal court’s conclusion was incorrect.
- We propose that informativeness in asserting scientific facts, rather than truthfulness, ought to be the gold standard for evaluating commercial speech about pharmaceuticals.

In May of 2015, a small Irish pharmaceutical manufacturer, Amarin, caused a major stir when it preemptively filed suit against the United States Food and Drug Administration (FDA) to ensure that it could distribute certain statements about its prescription pill Vascepa, composed of an esterified form of the omega-3 fatty acid eicosapentaenoic acid (EPA), derived from fish oil [1]. Vascepa had already been FDA-approved in 2012 to treat “very high” levels of triglycerides (>500 mg/dL), which was expected to prevent pancreatitis. However, Amarin wished to market the product to physicians for patients with moderate triglyceride levels (≥200 mg/dL and <500 mg/dL) to reduce those patients’ risk of cardiovascular disease (CVD)—an “off-label” indication that the FDA had not approved. Fearing that the FDA would treat this action as criminal misbranding, Amarin launched its lawsuit, and in August, a federal court agreed with Amarin, claiming that the First Amendment protected its commercial speech rights to make claims about its product that were “truthful and non-misleading” [2]. While this was not the first (or even the most recent) court case to undermine the FDA’s authority to regulate how pharmaceutical companies promote their products [3–5], it is unusual for its close examination of the essential truthfulness of the claims at issue.

Here, we argue that the central claim for which Amarin sought legal relief has essentially no truth content and is therefore only likely to serve the company’s economic interest on the presumption that it will be misinterpreted. We will then argue that even setting aside the vacuousness of this particular claim, Amarin’s interpretation of the scientific evidence that it considers to support its claim relies on a problematic circularity of reasoning.

## Fish Oil Derivatives and Cardiovascular Disease

In July 2009, while Vascepa was still in development, the FDA and Amarin established a formal agreement in which the FDA acknowledged that it would approve Vascepa as a treatment of moderately high triglyceride levels provided that Amarin completed a clinical trial demonstrating that Vascepa reduced persistently high triglyceride levels among statin-treated patients. This study, called ANCHOR, was initiated in 2009 and took approximately 4 years to complete. It showed that for patients already taking a statin, Vascepa produced significantly lower triglyceride levels relative to placebo [6].

While ANCHOR was underway, however, circumstances changed. Clinical trials involving the non-statin cholesterol-lowering drugs niacin [7,8] and fenofibrates [9] were completed and published—and each failed to show that reduction in lipid levels with these drugs correlated with a reduction in CVD. The FDA took these results to undermine the theoretical presuppositions of their prior agreement with Amarin and nullified its commitment to approve Vascepa on this basis—an action which it has only taken ten times in approximately 1,000 such agreements over the past 7 years [10].

Denied regulatory approval, Amarin nevertheless wished to continue promoting Vascepa for this indication off-label. It thus filed suit against the FDA to “ensure [the] ability to engage in truthful and non-misleading speech free from the threat of a misbranding action” [1]. At the center of their case was the “truthful and non-misleading”-ness of a single claim, which Amarin wished to distribute as part of their promotional materials:

C: Supportive but not conclusive research shows that consumption of EPA and docosahexaenoic acid (DHA) omega-3 fatty acids may reduce the risk of coronary heart disease.

The FDA argued that although C would be acceptable for promoting a dietary supplement, the statutory standards relating to prescription drugs are higher. Since Vascepa is marketed as a prescription drug, this statement was deemed to be “potentially misleading” about the strength of the evidence [2,10]. Nevertheless, according to the federal district court’s opinion, C was a “truthful” account of the existing state of scientific evidence, and the “supportive but not conclusive research” preface was unlikely to be misinterpreted by physicians [2].

## No Truth Content

We can agree with the court that C is in some sense truthful. However, it is not truthful because it is informative about the state of scientific evidence. Instead, it is truthful because it has almost no truth content—that is, the truth of C does not depend upon scientific facts. To illustrate, we can alter the language of C slightly:

C*: Supportive but not conclusive research shows that consumption of EPA and DHA omega-3 fatty acids may **or may not** reduce the risk of coronary heart disease.

This alteration makes explicit how the second clause is a tautology—it is trivially true because of its logical construction. A proposition of the form “P or not P” is always true, regardless of the state of scientific evidence. Logically speaking, C* asserts almost nothing about the state of scientific evidence. The “supportive but not conclusive” qualification is therefore a red herring vis-à-vis the truthfulness of C or C*. All this qualification really “says” is that non-conclusive research has been conducted, but then the second half of the claim expresses nothing at all about the clinical import of that research.

But the more important point is that the truth content of C does not differ from C*. Yet, C* is almost certainly less likely to persuade physicians that Vascepa is a clinically useful product. This suggests that Amarin’s economic interest can only be furthered by C being misleading. That is, for C to convey the “information” that the company wants it to convey—i.e., that Vascepa is a viable treatment option for reducing the risk of CVD—it needs to be trading off on the ambiguity between two states of affairs: (1) a state of evidence that has confirmed a causal relationship, but for which uncertainty still remains about whether or not this relationship is clinically significant; and (2) a state of evidence that has neither confirmed nor disconfirmed a causal relationship. The current state of scientific evidence concerning EPA is decidedly the latter of these two choices, which is made clearer in C* but is ambiguous in C.

## Circular and Inconsistent Reasoning

Setting aside the specific promotional statements that Amarin wished to make, the case here ultimately boils down to a difference in how the parties interpret the state of scientific evidence and, in particular, how the parties understand the truthfulness of another scientific claim:

X: Lowering moderate, persistent triglyceride levels reduces the incidence of CVD among patients already treated with a statin (and diet and exercise).

Amarin and the FDA agree that X is not yet known to be either definitively true or false, although, as noted above, the FDA believes there is now good reason to doubt the truth of X. Nevertheless, Amarin is arguing that they should be allowed to market Vascepa as if X is true. In other words, because X is not yet known to be false, then this is sufficient to make Amarin’s statement C—which amounts to saying that “X is possibly true”—truthful and non-misleading.

In justifying its position to the federal district court, Amarin also argued that physicians already use triglyceride-lowering drugs in combination with a statin for the off-label indication of interest [1,2]. That is, because physicians already behave as if X is true, then it should be acceptable commercial speech for Amarin to market a product using statements that assume X is true.

However, there is a problematic circularity of reasoning here. Physicians’ behavior concerning X is conditional on their belief in the truth of another, related claim:

Y: Reducing low-density lipoprotein (LDL) cholesterol with a statin reduces the incidence of cardiovascular events.

Y has been well confirmed by rigorous investigations, and there are now several statins on the market for which this relationship between the surrogate outcome (reducing LDL cholesterol) and the clinical outcome (incidence of cardiovascular events) holds [11]. The presumed truth of X is, in large part, based upon analogical reasoning from Y. The thought was that since the LDL cholesterol surrogate was predictive of the clinical outcome for statins, then perhaps other lipid-lowering mechanisms would also be predictive surrogates for CVD with other classes of drug.

This is a prima facie reasonable inference and was the basis for the original agreement between the FDA and Amarin. However, after the signing of that agreement, new evidence emerged casting doubt on the validity of this inference [12]. The relationship between lowering a lipid level and a corresponding reduction in CVD now does not appear to be robustly generalizable outside of statins [13]. In other words, what we might call the “boundary of uncertainty” about the different classes of lipid-lowering drugs and mechanisms for which the lipid–CVD relationship held was clarified somewhat by recent trials, and it now appears that EPA falls outside this boundary. Thus, the argument that physicians regularly recommend EPA and other lipid-lowering agents in addition to statins to reduce the risk of CVD is no longer an adequate justification to market EPA for this indication, since this practice ought to be conditional on the very same evidence that invalidated the original FDA–Amarin agreement.

In its court filings, Amarin responded to this objection by arguing that its drug is different from fenofibrates and niacin, the other two drug classes that had recently failed to uphold the lipid–CVD relationship [1]. Amarin pointed out that, in contrast to these other agents, there had been no direct tests of EPA that definitively show that lipid–CVD does not hold for it. Therefore, it is not scientifically accurate to place EPA outside the boundary of uncertainty.

This argument appears to have been decisive to the federal district court judge [2]. However, there is a second circularity of reasoning here: as explained above, the validity of the surrogate, and the plausibility of X, is based on analogical reasoning from Y. However, Y also refers to another class of drugs—the statins. The scientific plausibility of Amarin’s marketing campaign already depends upon indirect evidence from another class of agents. Thus, the company is trying to have it both ways: when evidence from another drug class supports its economic interest, it is happy to report and exploit the analogical argument to its advantage. But then when evidence from another class casts doubt on the efficacy of its agent, Amarin insists that analogical reasoning is invalid and therefore the evidence should not count against Vascepa. For this to be a sound argument, additional premises are needed to explain why EPA is more like a statin than a fenofibrate or niacin. In the absence of those premises, this argument is internally inconsistent.

## Discussion

This analysis reveals a number of important considerations about promotional speech for off-label drug marketing. First, there is a need to distinguish between “truthful claims,” on the one hand, and “informative claims,” on the other. Amarin’s statement about what may or may not be the case as concerns their product is certainly a truthful claim. But it is only vacuously so. It is a tautology, not a statement of empirical fact. A tautology is not misleading in itself; however, disguising a tautology, even as a weak claim about evidence, is misleading. We thus believe that “truthfulness” is not a sufficiently restrictive criterion for regulating promotional speech as concerns off-label medications. A better and clearer standard would demand that promotional claims must be informative, in the sense that they actually have empirical truth content, which is the assurance that FDA review and validation provides.

Second, even if Amarin’s claims cannot be considered fraudulent, we believe it would be unethical for a company to rely on misinterpretations of truth in marketing its products. Given how weak the evidence is for EPA having any cardiovascular benefits in the patient population sought by Amarin [14], it is also important to observe this potential for misinterpretation can only run in one direction—that is, physicians inferring that the evidence is stronger than it actually is.

Finally, if the possible truth of a scientific claim is sufficient to market a pharmaceutical off-label, then this would seriously undermine the authority of the FDA and potentially erode public trust in the regulatory system [15]. Trust in the medical research enterprise—and the regulatory system that oversees this enterprise—is conditional on the belief that medical products and practices have more than mere possibility of efficacy behind them [16]. In the modern era of evidence-based medicine, “supportive but not conclusive” evidence should be grounds for further research, not for changing clinical practice.

For all of these reasons, Amarin’s successful lawsuit providing it the ability to make truthfully-ambiguous claims when marketing its prescription drug products is troubling. There can certainly be disagreement over the state of evidence and the warrant of inferences. But this does not mean that anything goes. The FDA rescinded its agreement with Amarin because of a qualitative shift in the accumulating state of scientific evidence, transforming what would have been a valid scientific claim into an invalid one. This is unfortunate for Amarin, but uncertainty about how evidence will evolve is an intrinsic feature of the scientific enterprise. The truth must evolve accordingly.

## Abbreviations

CVD: cardiovascular disease
DHA: docosahexaenoic acid
EPA: eicosapentaenoic acid
FDA: United States Food and Drug Administration
LDL: low-density lipoprotein
